# Beta thalassemia prevention in India: evaluation of socio- cultural factors

**DOI:** 10.1186/1755-8166-7-S1-P123

**Published:** 2014-01-21

**Authors:** Swati Chawla, Rajnish Singh, VR Rao

**Affiliations:** 1 Department of Anthropology , University of Delhi, India

## Aim and background

There are approximately 240 million people across the world who are heterozygous for Beta thalassemia and 200,000 affected homozygous are born annually.

Prevention is a big challenge.

But the situation in India is different. Social, cultural, and religious issues are found to be closely intertwined with Beta thalassemia prevention. In spite of 10,000 annual Beta thalassemic births, the situation here is not well combated. Social stigma and negative attitude are largely understood to be the bounding factors.

The present study examines the prevention issue in the high risk community of India, analyzing their knowledge and perceptions in the light of available health models.

## Methods

A semi structured interview schedule was framed and implemented in the rural and urban (Delhi) population of a high risk community i.e. ARORAS and compared with the cosmopolitans (not at high risk).

## Results

Correct knowledge of carriers is a very important criteria, which is a major factor for the prevention of Beta thalassemia in a multi ethnic country like in India.

Participants were found to carry positive attitude towards the public perception of Beta thalassemia. In general, social discomfort were not a serious issue, but acceptance of life partner with Beta thalassemia trait was unacceptable among all the three populations but more among the rural Aroras which showed, lack of knowledge among them.

The acceptability for prevention strategies and its implication was high among all the three populations but rural Arora was found to be more proficient towards premarital screening(80.8%) and prenatal diagnosis(98.0%).

## Conclusion

A lack of knowledge about the disorder, its manifestations, survival rate, treatment availability, and psychosocial and cultural issues have created barriers to optimal health care including disclosure of Beta thalassemia status as well as to carrier testing. To overcome the problem there is an urgent need to have knowledge about people’s perception to find a common platform to harmonize various approaches for prevention.

